# Risk factors for mortality in patients undergoing continuous renal replacement therapy after cardiac surgery

**DOI:** 10.1186/s12872-021-02324-8

**Published:** 2021-10-21

**Authors:** Chang Liu, Hai-Tao Zhang, Li-Jun Yue, Ze-Shi Li, Ke Pan, Zhong Chen, Su-Ping Gu, Tuo Pan, Jun Pan, Dong-Jin Wang

**Affiliations:** 1 Department of Cardio-Thoracic Surgery, Nanjing Drum Tower Hospital Clinical College of Traditional Chinese and Western Medicine, Nanjing University of Chinese Medicine, Number 321 Zhongshan Road, Nanjing, 210008 Jiangsu China; 2 Department of Cardio-Thoracic Surgery, Nanjing Drum Tower Hospital, Peking Union Medical College, Chinese Academy of Medical Sciences, Graduate School of Peking Union Medical College, Beijing, 100010 China; 3 Department of Traditional Chinese Medicine, Nanjing Drum Tower Hospital, The Affiliated Hospital of Nanjing University Medical School, Nanjing, 210008 Jiangsu China; 4 Nanjing Drum Tower Hospital, The Affiliated Clinical College of Xuzhou Medical University, Nanjing, 210008 Jiangsu China; 5 Department of Cardio-Thoracic Surgery, Nanjing Drum Tower Hospital, Nanjing Medical University, Nanjing, 210008 Jiangsu China; 6 Department of Cardio-Thoracic Surgery, Nanjing Drum Tower Hospital, The Affiliated Hospital of Nanjing University Medical School, Nanjing, 210008 Jiangsu China

## Abstract

**Background:**

To investigate the risk factors for mortality in patients with acute kidney injury requiring continuous renal replacement therapy (AKI-CRRT) after cardiac surgery.

**Methods:**

In this retrospective study, patients who underwent AKI-CRRT after cardiac surgery in our centre from January 2015 to January 2020 were included. Univariable and multivariable analyses were performed to identify the risk factors for in-hospital mortality.

**Results:**

A total of 412 patients were included in our study. Of these, 174 died after AKI-CRRT, and the remaining 238 were included in the survival control group. Multivariable logistic regression analysis revealed that EuroSCORE > 7 (odds ratio [OR], 3.72; 95% confidence interval [CI], 1.92–7.24; p < 0.01), intraoperative bleeding > 1 L (OR, 2.14; 95% CI, 1.19–3.86; p = 0.01) and mechanical ventilation time > 70 h (OR, 5.03; 95% CI, 2.40–10.54; p < 0.01) were independent risk factors for in-hospital mortality in patients who had undergone AKI-CRRT. Our study also found that the use of furosemide after surgery was a protective factor for such patients (odds ratio, 0.48; 95% confidence interval, 0.25–0.92; p = 0.03).

**Conclusions:**

In summary, the mortality of patients with AKI-CRRT after cardiac surgery remains high. The EuroSCORE, intraoperative bleeding and mechanical ventilation time were independent risk factors for in-hospital mortality. Continuous application of furosemide may be associated with a better outcome.

## Background

Acute kidney injury (AKI) is a common complication after cardiac surgery, with an incidence of approximately 30% [1, 2]. Among patients who experience AKI, 2% to 5% require continuous renal replacement therapy (CRRT) [3, 4], and their mortality rate ranges from 50 to 80% [5, 6].

The identification of risk factors in cardiac surgery patients with AKI requiring CRRT (AKI-CRRT) may help in patient stratification, resulting in more appropriate resource utilization and improving patient prognosis [5, 7]. However, although many studies have investigated the risk factors for AKI in patients after cardiac surgery, few studies have focused on patients with AKI-CRRT [8, 9]. In existing research on the risk factors for AKI-CRRT, the sample size of the experimental group is small [8, 9]. Therefore, the purpose of this study was to assess the risk factors for mortality in patients who underwent AKI-CRRT after cardiac surgery.

## Methods

### Study design and settings

Ethical approval for this retrospective study was obtained from the Medical Ethics Committee of Affiliated Nanjing Drum Tower Hospital, Nanjing University Medical College (2020-281-01), and the study was conducted according to the principles of the Declaration of Helsinki.

We continuously included patients with AKI-CRRT in our centre from January 2015 to January 2020. A total of 412 patients met the eligibility criteria, including 174 patients who died in the hospital and 238 surviving patients included as the control group.

### Surgical techniques and cardiopulmonary bypass

Median sternotomy and cardiopulmonary bypass (CPB) were used for all patients. The ascending aorta was cannulated with a patient size-appropriate cannula. Venous cannulations were performed with separate cannulas in the superior and inferior venae cavae. The initial volume of the antegrade cold blood cardioplegia solution (4:1 cardioplegia solution to blood ratio) was twice the volume needed for the cessation of all cardiac electrical activity but never less than 1,000 m. Cardiac arrest was maintained by retrograde infusion of 300 mL of blood cardioplegia solution (8:1 cardioplegia solution to blood ratio) every 20 min. Occasionally, intermittent antegrade cold blood cardioplegia was used according to the surgeon’s preferences [10].

### Usage of CRRT and diuretics

The CRRT mode used in our study was continuous venous-venous hemodiafiltration (CVVHDF). During CRRT, patients were typically administered oral diuretics (furosemide and torsemide). The usual initial dose of furosemide was 20 mg, administered once daily. If needed, the same dose was administered 6 to 8 h later. The usual initial dose of torsemide was 10 mg, also administered orally once daily. If the diuretic response was not satisfactory, the dose of torsemide could be increased to 20 mg once daily.

### Diagnostic criteria of AKI-CRRT

1. Anuria (negligible urine output for 6 h); 2. Severe oliguria (urine output < 200 mL over 12 h); 3. Hyperkalaemia (serum potassium concentration > 6.5 mmol/L); 4. Severe metabolic acidosis (pH < 7.2 despite normal or low partial pressure of carbon dioxide in arterial blood); 5. Volume overload (especially pulmonary oedema unresponsive to diuretics); 6. Pronounced azotaemia (urea concentrations > 30 mmol/L or creatinine concentrations > 300 μmol/L); 7. Clinical complications of uraemia (e.g., encephalopathy, pericarditis, neuropathy) [11].

### Statistical analyses

IBM SPSS statistical software was used (Statistics for Windows, version 26, IBM Corporation, Armonk, NY, USA). The normality of the distribution of continuous variables was assessed by the Shapiro–Wilk test. Normally distributed variables are expressed as the mean ± standard deviation and were compared using Student’s t-test. Nonparametric continuous variables are expressed as medians (interquartile ranges (IQRs)) and were compared using the Mann–Whitney U test. Categorical data were compared using the chi-square test or Fisher’s exact test.

For additional analyses, all statistically significant variables in univariable analysis (P < 0.05) and variables considered by clinicians to be potentially significant were included in a multivariable logistic regression model to assess the independent associated postoperative mortality in patients with AKI-CRRT. Collinearity diagnostics were performed using tolerance estimates for individual variables in a logistic regression model.

## Results

### Patient characteristics

During the study period, 8477 patients were admitted to our centre, and the incidence of AKI-CRRT after cardiac surgery in our centre was approximately 4.9%, which is consistent with previous reports [12, 13]. Finally, 412 patients were included in our study. A total of 174 patients died after AKI-CRRT (mortality group), and the remaining 238 patients were included in the survival control group. No significant differences were found regarding sex, weight, New York Heart Association (NYHA) class, preoperative left ventricular ejection fraction, preoperative left ventricular end-diastolic diameter, or previous medical history between the two groups (P > 0.05). Compared with the control group, patients in the mortality group were older (61.59 ± 11.77 vs. 57.99 ± 12.34, P = 0.003). A higher EuroSCORE (7.11 ± 2.18 vs. 4.85 ± 2.37, P < 0.001) and evaluated pulmonary artery pressure (PAP; 49.78 ± 15.52 vs. 46.64 ± 13.49, P = 0.048) increased the mortality of patients with AKI-CRRT (Table 1).

**Table 1:** Preoperative baseline and characteristics among the mortality and control groups

Variable	Control (n = 238)	Mortality (n = 174)	p value
Age (year)	57.99 ± 12.34	61.59 ± 11.77	0.003
Gender (male, %)	160, 67	110, 63	0.398
Weight (kg)	64.26 ± 12.36	63.16 ± 10.52	0.343
*NYHA class (n, %)*			0.238
I	8, 3	6, 3	
II	66, 28	60, 34	
III	142, 60	92, 53	
IV	22, 9	16, 9	
EuroSCORE	4.85 ± 2.37	7.11 ± 2.18	< 0.001
*Echocardiogram variables*			
Preoperative LVEF (%)	30.84 ± 3.97	31.02 ± 4.03	0.640
Preoperative LVDd (cm)	7.04 ± 1.10	6.95 ± 1.03	0.374
Evaluated PAP (mmHg)	46.64 ± 13.49	49.78 ± 15.52	0.048
*Previous medical History (n, %)*			
Acute myocardial infarction	61, 26	39, 22	0.452
Atrial fibrillation	56, 24	39, 22	0.791
Diabetes mellitus	26, 11	20, 11	0.856
Chronic renal disease	4, 2	3, 2	0.937
Hypertension	65, 27	41, 24	0.390
Liver diseases	8, 3	6, 3	0.962
COPD	15, 6	4, 2	0.056
Cancer	3, 1	3, 2	0.698
Smoking	46, 19	35, 20	0.843
Excessive alcohol	20, 8	15, 9	0.938
Iodine contrast medium use	228, 96	169, 97	0.768
Vancomycin use	4, 2	2, 1	0.658
Gentamicin use	0	0	—
Others renal-injury drug	2, 1	5, 3	0.115

NYHA: New York Heart Association, COPD: Chronic obstructive pulmonary disease, LVDd: Left ventricular end-diastolic diameter, LVEF: Left ventricular ejection fraction, PAP: Pulmonary artery pressure, Means ± SD

### Intraoperative and postoperative variables between the mortality and survival control group

There were no significant differences in the type of cardiac surgery or the proportion of atrial fibrillation ablation and deep hypothermic circulatory arrest (DHCA) between patients. However, patients who died in the hospital had longer CPB (200.12 ± 61.70 vs. 180.93 ± 66.68, P = 0.003) and aortic cross clamp (ACC) durations (149.41 ± 50.15 vs. 130.74 ± 54.05, P < 0.001) (Table 2).

**Table 2:** Intraoperative and postoperative variables among the Mortality and Control groups

Variable	Control (n = 238)	Mortality (n = 174)	p value
*Intraoperative variables*			
Type of cardiac surgery (n, %)			0.657
CABG	43, 18	32, 18	
AVR	10, 4	7, 4	
MVR	86, 36	66, 38	
AVR + MVR	50, 21	40, 23	
CABG + valvular surgery	8, 3	3, 2	
David/Wheats/Bentall procedure	26, 11	21, 12	
Other aortic surgery	10, 4	5, 3	
Atrial fibrillation ablation (n, %)	40, 17	23, 13	0.318
CPB (minutes)	180.93 ± 66.68	200.12 ± 61.70	0.003
ACC (minutes)	130.74 ± 54.05	149.41 ± 50.15	< 0.001
DHCA (n, %)	10, 4	15, 9	0.063
Intraoperative bleeding (ml)	700 (400–1000)	900(600–1200)	0.001
RBC transfusion (n, %)			0.089
< 500 ml	53, 22	39, 22	
500–1000 ml	141, 59	79, 45	
ml	26, 11	48, 28	
> 1500 ml	18, 8	8, 5	
FFP transfusion (n, %)	47, 20	60, 34	0.002
Other colloid products (n, %)	22, 9	17, 10	0.857
*Postoperative outcomes*			
Drainage on the POD1 (ml)	40 (0–475)	205 (100–600)	< 0.001
Re-operation (n, %)	8, 3	17, 10	0.009
Septic shock (n, %)	0	8, 5	< 0.001
ECMO use (n, %)	1, 0.4	10, 6	0.001
IABP use (n, %)	5, 2	18, 10	< 0.001
Mechanical ventilation time (hours)	28 (23–34)	68 (63–74)	< 0.001
ICU stay time (days)	9.32 ± 2.50	20.55 ± 7.20	< 0.001
*Renal protection drugs*			
Furosemide (n, %)	196, 82	127, 73	0.061
Torasemide (n, %)	90, 38	49, 28	0.038
Tolvaptan (n, %)	63, 26	36, 21	0.175
Jin-Shui Bao capsule (n, %)	214, 90	146, 84	0.070

CABG: Coronary artery bypass grafting, AVR: Aortic valve replacement/repair, MVR: Mitral valve replacement/repair, CPB: Cardiopulmonary bypass, ACC: Aortic Cross Clamp, Mean ± SD / Median (interquartile range), DHCA: Deep hypothermic circulatory arrest, POD1: The first postoperative day, ARDS: Acute Respiratory Distress Syndrome, IABP: Intra-aortic balloon pump, RBC: Red blood cell, ECMO: Extracorporeal membrane oxygenation, ICU: Intensive care unit, FFP: Fresh frozen plasma

Although patients in the mortality group had more bleeding during the operation (median: 900 mL, IQR: 600–1200 mL vs. median: 700 mL, IQR: 400–1000 mL, P = 0.001) and received more fresh frozen plasma (FFP; 60 vs. 47 P = 0.002), there were no significant differences in the amount of red blood cells (RBCs; P = 0.089) or other colloid products (P = 0.857) transfused between the two groups (Table 2).

Patients in the mortality group had a greater drainage volume in the first 24 h after surgery (median: 205 mL, IQR: 100–600 mL vs. median: 40 mL, IQR: 0–475 mL, P < 0.001). Patients in the mortality group who underwent reoperation (17 vs. 8, P = 0.009), experienced septic shock (8 vs. 0, P < 0.001), and required extracorporeal membrane oxygenation (ECMO; 10 vs. 1, P = 0.001) and used of an intra-aortic balloon pump (IABP; 18 vs. 5, P < 0.001) were higher. Moreover, patients in the mortality group required use of mechanical ventilation for a longer time (median: 68 h, IQR: 63–74 h vs. median: 28 h, IQR: 23–34 h, P < 0.001) and required longer stays in the intensive care unit (20.55 ± 7.20 h vs. 9.32 ± 2.50 h, P < 0.001) (Table 2).

The proportion of patients in the survival group who used torsemide was higher (90 vs. 49, P = 0.038). However, the use of furosemide, tolvaptan or Jin-Shui Bao capsules was not different between the two groups (Table 2).

### Multivariable analysis between the mortality and survival control groups

The variables that were finally entered into the multivariable logistic regression included age > 70 years, EuroSCORE > 7, evaluated PAP > 55 mmHg, CPB > 240 min, DHCA, intraoperative bleeding > 1 L, intraoperative RBC transfusion, intraoperative FFP transfusion, drainage on POD1 > 500 mL, reoperation, ECMO use, IABP use, mechanical ventilation time > 70 h, postoperative furosemide, postoperative torasemide, and postoperative Jin-Shui Bao capsules. We found that EuroSCORE > 7, intraoperative bleeding > 1 L and mechanical ventilation time > 70 h were independent risk factors for in-hospital mortality in patients who underwent AKI-CRRT. The use of furosemide after surgery was a protective factor for these patients (odds ratio, 0.48; 95% confidence interval, 0.25–0.92; p = 0.03) (Table 3).

**Table 3:** Multivariable analysis of factors that may be associated with mortality

Variable	Odds ratio	95% Confidence Interval	p value
Age > 70 years	1.50	0.73–3.11	0.27
EuroSCORE > 7	3.72	1.92–7.24	< 0.01
Evaluated PAP > 55 mmHg	0.64	0.343–1.21	0.17
CPB > 240 min	0.83	0.41–1.67	0.60
DHCA	2.12	0.81–5.54	0.13
Intraoperative bleeding > 1 L	2.14	1.19–3.86	0.01
Intraoperative RBC transfusion	0.96	0.64–1.44	0.85
Intraoperative FFP transfusion	1.81	0.89–3.66	0.10
Drainage on the POD1 > 500 ml	0.85	0.46–1.58	0.61
Re-operation	1.11	0.27–4.54	0.88
ECMO use	2.12	0.14–31.35	0.58
IABP use	3.33	0.84–13.24	0.88
Mechanical ventilation time > 70 h	5.03	2.40–10.54	< 0.01
Postoperative furosemide	0.48	0.25–0.92	0.03
Postoperative torasemide	0.80	0.46–1.39	0.43
Postoperative jin-shui bao capsule	0.92	0.38–2.25	0.86

The continuous variables were converted to binary variables by upper quartile (≥ 75th percentile)

PAP: Pulmonary artery pressure, CPB: Cardiopulmonary bypass, ACC: Aortic Cross Clamp, DHCA: Deep hypothermic circulatory arrest, POD1: The first postoperative day, ECMO: Extracorporeal membrane oxygenation, IABP: Intra-aortic balloon pump; ICU: Intensive care unit

## Discussion

This retrospective study included more than 400 patients who required AKI-CRRT after undergoing cardiac surgery, representing approximately 4.9%. We found that a EuroSCORE > 7, massive intraoperative bleeding and prolonged mechanical ventilation time were independent risk factors for mortality in patients with AKI-CRRT, while continuous use of furosemide may be associated with a better outcome.

The EuroSCORE II is widely used to predict mortality after cardiac surgery [14]. Halpin and colleagues reported that the EuroSCORE II had better predictive discrimination for operative mortality than the EuroSCORE I and the Society of Thoracic Surgeons (STS) risk score [15]. Interestingly, David and colleagues also reported that the EuroSCORE could be used to predict postoperative AKI [16]. Moreover, Drosos and colleagues suggested that patients with a higher EuroSCORE II were more likely to require renal replacement therapy after cardiac surgery (7.8 ± 14.4 vs. 1.9 ± 2.1, P = 0.014) [17]. In our study, the EuroSCORE II values of deceased patients were higher, which is in line with previous experiences.

Intraoperative bleeding may be affected by patient- and surgery-related factors, such as the use of anticoagulants, systemic inflammatory responses, the consumption and dilution of clotting factors, and fibrinolysis associated with the use of CPB [18]. Among them, approximately 15%-20% of patients have excessive bleeding during cardiac surgery and consume approximately 80% of blood products used in cardiac surgery procedures [19]. Haemodynamic instability caused by excessive blood loss is an important cause of AKI [20], and the mortality of patients after surgery is also significantly increased [21]. In our centre, Stanford type A aortic dissection patients undergoing emergency surgery account for 15% of all cardiac surgery patients. Therefore, the amount of surgical blood loss and the use of AKI-CRRT in our centre are relatively high.

In addition to the EuroSCORE II, massive intraoperative bleeding was another risk factor for mortality in patients with AKI-CRRT. Bleeding is a determinant of excessive haemodilution during cardiac surgery, and the lowest haematocrit is widely recognized as related to AKI [22–25]. The main explanation for this association is that first, the increase in renal tubular transport leads to an increase in kidney energy requirements. Second, the reduced oxygen delivery caused by haemodilution can exacerbate renal ischaemic dysfunction, especially in the renal medulla, which is sensitive to oxygen [20, 21, 24]. Unlike intraoperative bleeding, the impact of blood transfusion on AKI after cardiac surgery remains controversial. Some studies contend that blood transfusion worsens CPB-initiated systemic inflammatory response syndrome with a second inflammatory response, thereby increasing the incidence of AKI [26]; in contrast, Ng et al. reported that blood transfusion did not play a role in influencing AKI outcomes [25]. In our study, multivariable regression analysis showed that blood transfusion was not related to the mortality of patients with AKI-CRRT after cardiac surgery. Considering that low haematocrit increases the incidence of AKI and patient mortality, we recommend that blood transfusions be properly used to avoid excessive haemodilution.

Systemic inflammatory response syndrome caused by CPB often leads to AKI and acute lung injury that requires positive-pressure mechanical ventilation (MV) [27, 28]. There is a complicated internal connection between kidney and lung organ dysfunction, broadly categorized as haemodynamic and neurohormonal [27]. Multiple systematic reviews have shown that MV can greatly increase the incidence of AKI in critically ill patients [28, 29]. In addition, Fuhrman et al. reported that AKI was associated with a longer duration of MV after cardiac procedures (OR, 1.47; 95% CI: 1.15–1.89) [30]. Therefore, we suggest that it may be beneficial to withdraw MV as soon as possible for patients undergoing AKI-CRRT, but further research and verification are needed to support this claim.

Multivariable regression analysis showed that continuous use of furosemide after surgery was a protective factor that may reduce the mortality of patients with AKI-CRRT. For AKI-CRRT patients, the value of diuretics remains controversial. A recent large-scale cohort study supports our conclusion, however, suggesting that the difference in evaluation criteria was the cause of the controversy over the value of diuretics. The study authors reported that continuous infusion of furosemide increased urine output and helped with successful CRRT discontinuation [31]. However, a previous systematic review reported that furosemide is not associated with significant clinical benefits on renal outcome, including in-hospital mortality and renal replacement therapy or dialysis [32]. In addition, Vargas et al. suggested that torsemide might show a better dose-dependent diuretic effect in patients after CRRT treatment [33]. In our research, both torsemide (P = 0.038) and the traditional Chinese medicine Jin-Shui Bao capsule (P = 0.070) showed potential value in protecting kidney function, but both need to be verified by specially designed clinical trials.

## Limitation

Some limitations should be considered in this study. First, because of the retrospective study design, some predictors of AKI prognosis, such as fractional excretion of urea, the furosemide stress test and creatinine clearance assessed by 6-h urine, were not included. Second, also related to the retrospective nature of the study, many confounding factors may have affected the reliability of our conclusions, and our research suggests that oral furosemide during AKI-CRRT after cardiac surgery may be beneficial, but further research is necessary. Third, in the multivariable regression analysis, continuous variables were converted to binary variables by upper quartile (> 75th percentile), which may lead to partial loss of information. Finally, the data come from a single centre, and agency-specific variables may have influenced the current results. Future studies will focus on improving the generalization and practicability of the research results.

## Conclusion

In summary, the mortality of patients with AKI-CRRT after cardiac surgery remains high. The EuroSCORE, intraoperative bleeding and mechanical ventilation time were independent risk factors for in-hospital mortality. Continuous use of furosemide may be associated with a better outcome.

## Data Availability

The datasets used and/or analysed during the current study are available from the corresponding author on reasonable request.
